# Live yeasts accelerate *Drosophila melanogaster* larval development

**DOI:** 10.1242/jeb.247932

**Published:** 2024-10-04

**Authors:** Yanira Jiménez-Padilla, Babafemi Adewusi, Marc-André Lachance, Brent J. Sinclair

**Affiliations:** Department of Biology, University of Western Ontario, London, Ontario N6A 5B7, Canada

**Keywords:** Gut yeasts, Development time, Insect symbionts, Gnotobiotic, Microbiome, Gut–brain axis

## Abstract

Insect guts house a complex community of microbes that affect host physiology, performance and behavior. Gut microbiome research has largely focused on bacteria–host symbioses and paid less attention to other taxa, such as yeasts. We found that axenic *Drosophila melanogaster* (reared free of microbes) develops from egg to adult more slowly (ca. 13 days) than those with a natural microbiota (ca. 11.5 days). Here, we showed that live yeasts are present and reproducing in the guts of flies and that the fast development time can be restored by inoculating larvae with a single yeast species (either *Saccharomyces cerevisiae* or *Lachancea kluyveri*). Nutritional supplements (either heat-killed yeasts, or a mix of essential vitamins and amino acids) slightly sped the development of axenic flies (to ca. 12.5 days), but not to the same extent as live yeasts. During the first two instars, this acceleration appears to result from additional macronutrient availability, but during the third instar, when most growth occurs, live yeasts increased feeding rate, implying an effect mediated by the gut–brain axis. Thus, the fly–yeast interaction extends beyond yeasts-as-food to yeasts as beneficial interactive symbionts.

## Teaser

**Highlighted Article:** One species of live yeast in the gut will support normal-speed *Drosophila* larval development. The yeasts must be alive, suggesting that yeasts are not just food, but have a biological interaction with their host.

## INTRODUCTION

Insects form persistent associations with microbes ranging from bacteria and archaea to fungi and protists (Douglas, 2015; Gurung et al., 2019). Most microbes are in the gut, and can affect phenotypes from digestion and nutrition (Engel and Moran, 2013; Ceja-Navarro et al., 2015), to metabolism (Wong et al., 2014), immunity (Jarosz, 1979; Dillon et al., 2005), development (Shin et al., 2011) and behavior (Dillon et al., 2000; Matsuura, 2001). Most microbiota studies focus on bacteria, while other taxa are understudied (Broderick and Lemaitre, 2012; Hoang et al., 2015; Gurung et al., 2019). *Drosophila melanogaster* has a simple gut microbial community dominated by three bacterial taxa (Acetobacteraceae, Enterobacteriaceae and Lactobacillales) (Chandler et al., 2011; Wong et al., 2011), and the yeast genera *Saccharomyces*, *Hanseniaspora* and *Pichia* (Phaff et al., 1956a; Lachance et al., 1995; Chandler et al., 2012). Although the impact of bacteria on host phenotype is well explored, the effect of yeasts is usually attributed to their macronutritional content rather than a biotic interaction (Anagnostou et al., 2010; Broderick and Lemaitre, 2012; Hoang et al., 2015; Le Bourg et al., 2015; Meshrif et al., 2016; Grangeteau et al., 2018; McMullen et al., 2020; Mure et al., 2023).

Many *Drosophila* species, including *D. melanogaster*, feed and oviposit in yeast-rich decaying fruits (Starmer, 1981), and flies are preferentially attracted to volatiles from the yeast species found in their gut (Becher et al., 2012; Hoang et al., 2015; Scheidler et al., 2015). Digesting yeasts provides the insect with amino acids, B-vitamins, sterols and fatty acids (Anagnostou et al., 2010), and the yeast complement changes over time as the fruit rots (and the microbial community develops; Mure et al., 2023). In the laboratory, commercial *Saccharomyces cerevisiae* is usually provided to *Drosophila* as food and to stimulate oviposition (Markow and O'Grady, 2006), even though *S. cerevisiae* is rarely recovered from flies in nature (Hoang et al., 2015). Flies provided with yeast develop faster (Anagnostou et al., 2010; Grangeteau et al., 2018; McMullen et al., 2020; Mure et al., 2023), produce more offspring (Simmons and Bradley, 1997), live longer (Skorupa et al., 2008) and have more robust immunity (Vass and Nappi, 1998). Yeasts survive and multiply in the *Drosophila* digestive tract (Giglioli, 1897; Reuter et al., 2007; Coluccio et al., 2008), although it is unclear whether they progress past the crop as vegetative cells or only as spores (Giglioli, 1897; Shihata and Mrak, 1951; Reuter et al., 2007; Coluccio et al., 2008). Nevertheless, although many gut microbe effects are thought to derive from microbial activity in the gut (for discussion, see Schmidt and Engel, 2021), the presence of live cells anywhere in the gut holds the potential for a biological (e.g. signaling) interaction between the yeast cell and its host. However, because dead (‘inactive’) *S. cerevisiae* is sufficient to maintain flies in the laboratory, yeasts have received little attention as living gut microbiota (Broderick and Lemaitre, 2012; Douglas, 2018; Gurung et al., 2019). Thus, only a few studies have explored how naturally occurring yeast symbionts affect their *Drosophila* hosts (Anagnostou et al., 2010; Hoang et al., 2015; Fischer et al., 2017; Murgier et al., 2019; Tang et al., 2019; McMullen et al., 2020; Mure et al., 2023).

Development time drives adult body size, fecundity and viability in *Drosophila* (Nunney, 1996), with cascading effects on generation time, population growth and competitive interactions with conspecifics (Kohane, 1994; Horváth and Kalinka, 2016). Gut microbes affect *D. melanogaster* development time: flies deprived of their gut microbes develop more slowly than conventionally reared flies (Shin et al., 2011; Storelli et al., 2011; Wong et al., 2014). Many microbiota studies use axenic (no microbiome) and gnotobiotic (known microbial complement) flies (Douglas, 2018), but most of these studies have focused on bacteria (Storelli et al., 2011; Wong et al., 2014; Consuegra et al., 2020; McMullen et al., 2020). However, yeasts do have an impact: live *Hanseniaspora uvarum* and *Kazachstania humilis* accelerate egg–adult development relative to axenic flies (McMullen et al., 2020; Mure et al., 2023). Furthermore, Mure et al. (2023) report subtle differences among yeast species: species such as *H. uvarum* and *K. humilis* that are present early in the fruit rotting process in nature had more of an effect than those – such as *Pichia kluyveri* – that become dominant in the fruit microbial community later. These effects appear to depend on properties of the yeast when it is alive: larvae develop faster than their axenic counterparts if fed high concentrations of heat-killed yeasts of any species, putatively because their internal nutrients are now available (Mure et al., 2023). Finally, egg–pupa development (but not pupation time) is accelerated in microbiota-replete flies supplemented with live *S. cerevisiae*, but less so with dead yeast (Grangeteau et al., 2018), suggesting that live yeasts may interact biologically with their host.

The potential mechanisms by which gut yeasts could influence growth and development (beyond simply providing nutrients) are uncertain. Gut yeasts probably reshape metabolism: *D. melanogaster* adults that were reared on live yeasts reprioritized energy allocation from carbohydrates to lipids (male) or protein (female) and larval transcriptomes indicate that larvae fed live yeasts express enriched gene sets associated with metabolic processes and nucleotide and protein processing (McMullen et al., 2020). Live yeasts may also provide specific nutrients – both branched-chain amino acids (Mure et al., 2023) and short chain fatty acids (McMullen et al., 2020) have been identified as candidate molecules.

Mure et al. (2023) and McMullen et al. (2020) concluded that the yeast–fly interaction is largely nutritional. However, for some time we have been conducting experiments that suggest the opposite: at least some of the impact of yeasts on fly development transcends nutrition to some sort of signaling relationship. Because of methodological variation in all of these yeast–fly studies, identifying when live yeasts affect development and under what circumstances is essential to understanding these discrepancies. Here, we showed that live yeasts accelerate *D. melanogaster* growth and development beyond their macronutritional value. We confirmed that *S. cerevisiae* and *Lachancea kluyveri* (first isolated from *Drosophila* spp. guts; Phaff et al., 1956b) are alive and reproducing in the *D. melanogaster* gut. Finally, we demonstrated that live yeasts supplied to axenic larvae in the second instar reduce development time by accelerating the development of third instar larvae – an effect that cannot be replicated through nutritional supplementation, and that could have been overlooked in previous experiments. Thus, although they clearly contribute nutritionally to larval development, yeasts play additional roles in the *D. melanogaster* gut microbiome by interacting directly with their animal hosts at critical points to determine host phenotypes.

## MATERIALS AND METHODS

### Experimental animals

We used outbred, wild-type *Drosophila melanogaster* Meigen 1830 collected from London, Ontario, Canada (43.00°N, 81.25°W) in 2008 (Marshall and Sinclair, 2010), and treated with tetracycline in 2012 to remove any *Wolbachia* (Salehipour-Shirazi et al., 2017). We reared flies on banana food [1 liter dH_2_O, 7.25 g agar, 27.5 g dry active yeast (Fleischmann's Yeast, Farinex, QC, Canada), 2 g methylparaben, 137.5 g bananas, 47.5 g corn syrup, 30 g liquid malt, 3 ml propionic acid (Markow and O'Grady, 2006)] at 21.5±1°C, 60±5% relative humidity and a 13 h:11 h light:dark photoperiod. We maintained flies in 35 ml vials (ca. 50 flies per vial), and maintained outbreeding by mating them in 3.7 liter plastic population cages (ca. 750 adults; see Sinclair et al., 2007). In the cages, we provided the flies with a Petri dish of banana food cut into slices to facilitate oviposition on vertical surfaces for egg collection 24 h later. We controlled for parental age and rearing density by transferring approximately 50 eggs per vial into 35 ml vials containing 10 ml Tucson fly food [1 liter dH_2_O, 15 g dry active yeast, 43 g sugar, 27 g cornmeal, 10 g agar, 4 ml propionic acid (adapted from Markow and O'Grady, 2006)], and collecting eggs from these synchronized flies.

To explore the role of insulin signaling in our phenotype, we used a *dilp2* knockout mutant (*dilp2^1^*, BDSC 30881) and a *chico* hypomorphic mutant (*chico^KG^*, BDSC 14337) from the Bloomington *Drosophila* Stock Center. We treated them with tetracycline for three generations to remove any *Wolbachia* (Hoffmann, 1988), reared them and collected eggs as described for our wild-type flies. At least five generations elapsed between the tetracycline treatment and our experiments.

Every experiment included *n*=10 vials, each containing five sterile eggs (see below) per treatment. We repeated each experiment three times (‘cohorts’), a minimum of 3 weeks apart, and with each cohort derived from a different parental generation.

### Rearing axenic and gnotobiotic *D. melanogaster*

We made axenic flies by surface-sterilizing eggs and growing them in sterile vials. We transferred approximately two hundred 5- to 10-day-old adult flies to oviposition cages (diameter=3.5 cm, height=5.8 cm) capped with a Petri dish of apple juice agar (100 ml fruit juice, 100 ml dH_2_O, 4 g agar) topped with a paste of inactive yeast (Flystuff 62-107, Gennessee Scientific, El Cajon, CA, USA) and distilled water. We allowed the females to feed for 3 days and then allowed females to lay eggs for 3–5 h on fresh apple juice agar without yeast paste. To sterilize eggs, we transferred batches of five eggs onto an autoclaved nylon filter (24 mm diameter, 20 μm pore, NY2002500, Millipore-Sigma, Oakville, ON, Canada), and surface-sterilized them by submerging them in 70% ethanol for 3 min, followed by a rinse in sterile phosphate-buffered saline (PBS; P4417, Millipore-Sigma). Preliminary work showed that five eggs maximized the success of our surface-sterilization. We inverted the filter to transfer the eggs to a thin layer of sterile yeast–sugar agar (1.5 g agar, 1.5 g active yeast. 4.3 g sugar, 100 ml dH_2_O), which we moved to a treatment vial containing 10 ml of sterile modified Tucson fly food (we excluded propionic acid, which inhibits fungal growth). We inoculated each vial with 10 µl of a treatment solution (described below) and plugged them with tight-fitting cellulose acetate plugs and paper caps to prevent contamination. We incubated these vials under standard growth conditions until adult emergence.

**Fig. 1.**
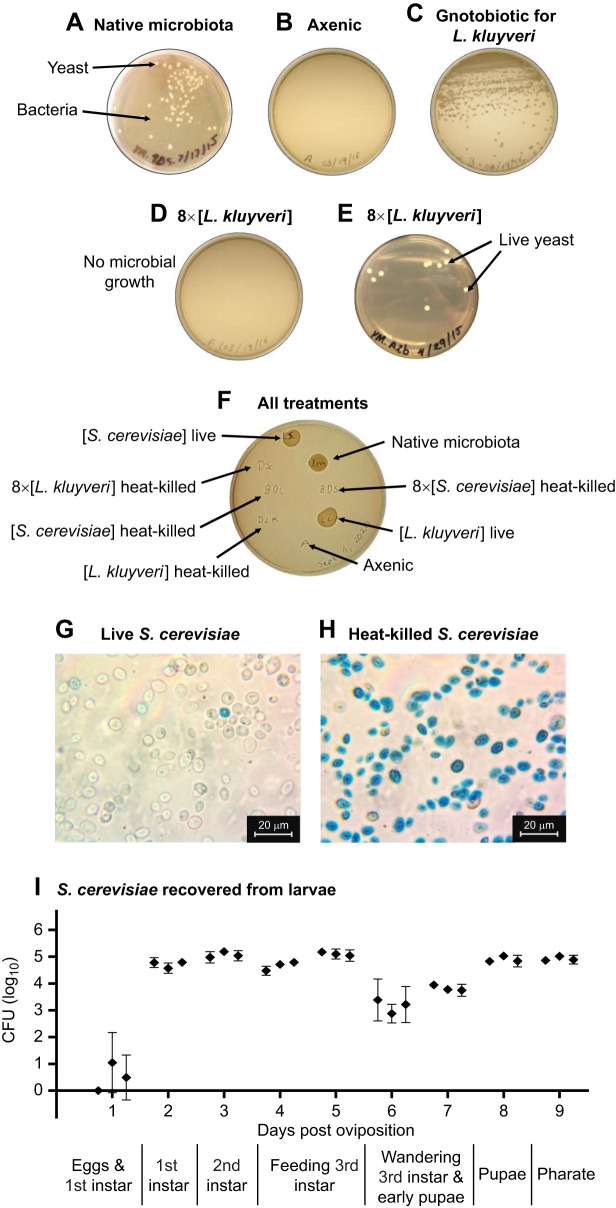
**Assessment of microbial state from all treatments at the beginning and end of each experiment.** Examples plates from various experiments demonstrating characteristics used to corroborate the state of each treatment. Data from treatments that showed unexpected microbial growth were discarded. For pilot experiments, we used a whole plate per treatment: (A) bacteria and yeast from native microbiota, (B) axenic, (C) live yeast, (D) heat-killed yeast, and (E) unexpected yeast growth in a heat-killed yeast treatment. (F) For all subsequent experiments, we plated 10 µl of each treatment in a spot test to assess microbial growth. (G) Live *Saccharomyces cerevisiae* and (H) heat-killed *S. cerevisiae* after 1 min exposure to 1% Methylene Blue. Dead yeast cells are blue whereas live cells (with intact membranes) remain colorless. Light field microscopy (100× objective lens with oil immersion). (I) Colony forming units (CFU) of *S. cerevisiae* from larvae and pupa. The developmental stage at which individuals (3 larvae or pupae per vial, 3 vials per day) were homogenized and plated is indicated below the *x*-axis.

We confirmed that this method generated axenic flies (Fig. 1). From each vial in our preliminary experiments (50 eggs per vial), we plated onto separate yeast-malt (YM) agar plates (YM agar: 1% w/v glucose, 0.5% peptone, 0.3% malt extract, 0.3% yeast extract, 2% agar): (1) three homogenized male flies; (2) three homogenized female flies; and (3) ca. 3 µl of food scraped from the food surface (all homogenized in 200 µl sterile PBS). We incubated these plates at 25°C and checked for visible bacterial or yeast colonies after 48 h and 7 days. We repeated this preliminary experiment for five different vials in each of three cohorts in addition to ca. 15 cohorts of preliminary experiments (data in Jiménez-Padilla, 2016). No bacterial or yeast colonies were observed on these plates after incubation. As part of preliminary experiments, we used PCR to detect any bacterial or archaeal DNA in axenic flies (see Supplementary Materials and Methods).

Nevertheless, for every vial, once flies had emerged after each experiment, we homogenized one male, one female and a 3 µl food scraping together in 100 µl sterile PBS, and spot-plated 10 µl of the homogenate on YM agar. We excluded data from any vials from which unexpected microbial colonies grew. In total, we excluded 222 of 3579 vials (6%) across the entire study.

### Yeast cultures

We obtained *L. kluyveri* (strain NRRL Y-12651) and *S. cerevisiae* (strain UWOPS 92-222.2) from the Department of Biology, University of Western Ontario yeast collection. For microscopy, Dr P. Lajoie (Department of Anatomy and Cell, University of Western Ontario) provided *S. cerevisiae* (strain W303) expressing green fluorescent protein (GFP). We stored the cultures at −80°C in Microbank bead vials (Pro-Lab Diagnostics, Richmond Hill, ON, Canada) and renewed our working cultures (on YM agar plates at 15°C) from frozen cultures every 2–3 months. We prepared yeast for treatments by inoculating YM agar plates with material from three colonies from a working culture plate and incubating them at 25°C for 3 days.

To prepare the live yeast treatment vials, we inoculated sterile Tucson food with 10 µl of a 1.3×10^8^ cells ml^−1^ suspension. We prepared the yeast suspensions by removing colonies from the YM agar with an inoculation loop and fully mixing them in 1 ml sterile PBS. We diluted the suspensions to determine concentration using standard absorbance curves (560 nm wavelength, 1 cm light path) calibrated to number of cells.

We generated heat-killed yeasts by preparing a series of cell suspensions at multiples of the 1.3×10^8^ cells ml^−1^ concentration we used for live yeasts. We killed these yeasts by incubating at 60°C for 10 min (1× and 2× concentrations), 20 min (4× concentration), 30 min (8× concentration) or 45 min (16×, 32× and 100× concentrations) with aspiration each 10 min to prevent pelleting. We previously determined these as minimum times to reliably yield no growth when plated on YM agar (Fig. 1D). We cooled heat-killed yeast suspensions for 30 min at room temperature before inoculating 10 µl in each vial. Neither *S. cerevisiae* nor *L. kluyveri* exposed to these treatments had visibly disrupted cell walls (Fig. 1G,H), and we confirmed that heat-killed yeast cells accumulated Methylene Blue (0.1% w/v mixed 1:1 with the yeast suspension and incubated for 1 min).

### Treatment groups

We plated all treatment suspensions in YM agar to confirm the sterility of PBS and heat-killed yeasts, active growth of live yeast cultures, and mixed microbial growth for the native microbiota treatment (Fig. 1). We discarded any treatments for which we identified unexpected growth (e.g. in heat-killed or sterile cultures), no growth when expected (e.g. live yeasts), or obvious microbial contamination (e.g. molds, bacteria). We used the following six treatments.

#### Axenic

Sterile eggs transferred to food, inoculated with 10 µl sterile PBS.

#### Native microbiota

We reintroduced the native microbiota of our fly colony by inoculating the food with homogenized conventionally reared flies (three adult flies in 200 µl of sterile PBS). Development time of these flies did not differ from the development time in our conventionally reared colony (Fig. S1A). Plating the homogenate on YM agar yielded several yeast species (including genera such as *Pichia*, which is not included in our experiments; data not shown), mold and bacterial colonies (Fig. 1A).

#### Gnotobiotic (*S. cerevisiae* or *L. kluyveri*)

We transferred sterile eggs to food inoculated with live yeast suspension (described above). We confirmed gnotobiotic state by observing yeast growth in YM plates inoculated with fly homogenates (Fig. 1C).

#### Heat-killed yeasts

Sterile eggs transferred to food inoculated with heat-killed yeast suspensions.

#### Heat-killed yeasts mixed in food

Larvae burrow into the food and live yeasts might reproduce and move within the food (but heat-killed yeasts should not). Furthermore, there is the possibility that the nutrients from a single yeast addition to the surface could be consumed (cf. Keebaugh et al., 2019). Thus, we made heat-killed yeasts available at different concentrations throughout the food column. We prepared these vials by mixing 50 µl heat-killed yeast in the food before dispensing or by pipetting 10 µl heat-killed yeast solution every 5 mm between five layers of food.

#### Nutritional supplements

We transferred sterile eggs to Tucson food supplemented with 20 essential amino acids and seven B-vitamins (Toshima and Tanimura, 2012; Wong et al., 2014). We prepared a filter-sterilized (0.45 µm pore size nylon syringe filter, ThermoFisher, Burlington, ON, Canada) solution of B-vitamins and amino acids in PBS, and mixed it into (still liquid) autoclaved food cooled to 55°C under aseptic conditions. The supplemented diet contained thiamine (1.4 mg l^−1^), riboflavin (0.7 mg l^−1^), nicotinic acid (8.4 mg l^−1^), pantothenate (10.8 mg l^−1^), pyridoxine (1.7 mg l^−1^), biotin (0.1 mg l^−1^), folic acid (9 mg l^−1^), arginine (210.7 mg l^−1^), aspartic acid (232.9 mg l^−1^), glutamic acid (294.2 mg l^−1^), tyrosine (45.3 mg l^−1^), tryptophan (510.5 mg l^−1^), alanine (445.5 mg l^−1^), asparagine (750.7 mg l^−1^), cystein (606.0 mg l^−1^), glutamine (730.8 mg l^−1^), glycine (375.5 mg l^−1^), histidine (958.5 mg l^−1^), isoleucine (655.9 mg l^−1^), leucine (655.9 mg l^−1^), lysine (913.5 mg l^−1^), methionine (746.0 mg l^−1^), phenylalanine (826.0 mg l^−1^) and proline (575.5 mg l^−1^).

### Viability and persistence of yeast cells in the *D. melanogaster* gut

We confirmed that yeast cells were alive and reproducing inside the gut of larvae in our gnotobiotic treatments. We placed axenic eggs (10 eggs per vial; 10 vials per yeast) into Tucson food vials inoculated with live *S. cerevisiae*, GFP-*S. cerevisiae* or *L. kluyveri*. We randomly selected three vials from each yeast species or strain and removed feeding third instar larvae (identified by their large size and confirmed by checking a subset of mouthparts as per Demerec, 1965). We surface-sterilized one larva per vial by submerging it in 70% ethanol for 1 min and rinsing with sterile PBS. We wet-mounted them on a microscope slide with Carolina observation gel (132700, Carolina, Burlington, NC, USA) to avoid crushing the specimens for whole-animal and intact gut images. We then removed the larvae and dissected the guts, which we surface-sterilized and wet-mounted on a slide where we crushed them using a sterile coverslip. We mounted and dissected pupae from yeast-inoculated vials as above, except we omitted the GFP-*S. cerevisiae*. After imaging, we removed the mounted guts under sterile conditions and plated them on YM agar to confirm the presence of live yeasts.

To determine the presence and structural integrity of the yeast cells, we took micrographs of larvae and pupae (and their guts) using phase-contrast (100× objective lens with oil immersion), and larvae and their guts using a combination of fluorescence and differential interference contrast (Axio Imager Z1 with ZEN 2012 software, Zeiss, Toronto, ON, Canada). Live yeast cells maintain their shape, while dead yeasts are visible as spheroplasts (Kelly and Nurse, 2011), and in phase-contrast, live and dead yeast cells have different refractive indices (Wiemken et al., 1970). We visually identified freshly budded yeast cells and ascospore production (Coluccio et al., 2008) to identify replication.

Yeasts were present in gnotobiotic larvae throughout development (Fig. 1I). After inoculation of the gnotobiotic vial, we collected three individuals per vial (3 vials per day throughout larval and pupal development) each day until eclosion. We sterilized these larvae and pupae as above and individually homogenized them in 100 µl of sterile PBS. We plated the diluted homogenates on YM agar and counted colony-forming units (CFU) after 48 h of incubation at 25°C.

### Development time

We prepared 12 vials for each treatment to account for possible contamination. If there was no contamination, we randomly selected 10 vials (using random.org) and discarded the others. In preliminary experiments, we saw no pupariation until 6 days post-oviposition, so after day 6 we counted the number of pupae and in each vial at 4 h intervals from lights-on at 07:00 h until 23:00 h (2 h after lights-off). We continued checking until no new adult flies had eclosed for 72 h. When we weighed individuals (MX5 microbalance, accurate to 1 µg, Mettler Toledo, Columbus, OH, USA), we did so 2 days post-pupariation for pupae and within 12 h of eclosion for adults; to calculate dry mass, we weighed after 48 h at 60°C. For experiments when the larvae had access to yeast on different days, we prepared a large group of axenic vials and randomly selected 10 vials per day for inoculation with live yeast and recorded pupariation time as described above.

### Developmental stages

To identify developmental stages, we prepared vials (*n*=5 eggs per vial, two vials per treatment per day; repeated four times) with native microbiota, axenic, live *S. cerevisiae* and heat-killed *S. cerevisiae* (8×). Each day after day 2 until all flies in a vial had pupariated we sampled food to assess microbial growth, measured dry mass of 1–3 individuals, and wet-mounted 1–2 larvae, which we aged according to Demerec (1965). We measured development time (as above) in five vials per treatment.

### Feeding rate

To quantify feeding rate of third instar larvae, we prepared vials (*n*=5 eggs per vial; 10 vials per treatment, but only one cohort) with native microbiota, axenic, live *S. cerevisiae* and heat-killed *S. cerevisiae* (8×). We selected five vials at random and removed a small portion of food and three larvae. We homogenized the food in 2 ml sterile water, and divided it into approximately three parts, each of which was placed in a Petri dish (3.5 cm diameter). We waited 1 min for the larvae to equilibrate before counting feeding lunges of each individual for 1 min, repeated three times with 30 s intervals in between (after Sewell et al., 1974). For each individual, we calculated the mean of these three 1-min trials, and used this value in analyses, pooling the vials to yield *n*=15 individuals per treatment.

### Statistical analyses

We performed all analyses using GraphPad Prism (Windows version 9.5.1, GraphPad, San Diego, CA, USA).

We used the 80% development time (hereafter ‘development time’) for each vial as a biological replicate for parametric analyses (Jakobs et al., 2015). To compare pupation times among treatments, we noted pupariation and eclosion for each individual (larvae pupate on the vial walls, so we marked each individual on the outside of the vial), and calculated the mean pupation time for each vial, and the duration of pupation for each individual. We compared development time and mass among treatments and cohorts using a two-way ANOVA followed by Tukey's *post hoc* tests. When we did not include cohort in an analysis, we compared development time, pupation time and pupation duration using one-way ANOVA. We analyzed lower-resolution data sets where we recorded pupation time daily (rather than hourly) using unequal variance Welch's ANOVA followed by Dunnet's T3 multiple comparisons test.

Preliminary analyses showed that neither formal survival analyses nor including cohort as a random factor affected our conclusions, so we conducted all our analyses using one-way ANOVA on pooled development times (treating the 10 vials in each cohort as a dataset with *n*=30 vials). For survival analyses, we used a log-rank (Mantel–Cox) analysis to make pairwise comparisons among treatments and determine cohort effects (Fig. S1, Table S2), followed by a table-wide Benjamini–Hochberg false discovery rate (FDR) correction for multiple comparisons. We present survival curves to match the 80% development time data in Fig. S1. We provide the data for all experiments separated by cohort (and formatted for survival analyses) in Dataset 1.

We examined the relationship between development time and dry mass with a Pearson product-moment correlation for each treatment followed by a table-wide FDR correction.

We compared larval feeding rates among treatments with one-way ANOVA followed by Tukey's *post hoc* tests.

## RESULTS AND DISCUSSION

### Live gut yeasts reduce *D. melanogaster* development time

We reared axenic and gnotobiotic *D. melanogaster* from egg to adult. Live yeast cells (including budding cells) were present in the guts of gnotobiotic larvae and pharate pupae (data not shown). Yeast spores have previously been reported to survive gut passage in adult *D. melanogaster* (Reuter et al., 2007) and vegetative cells have been recovered from adult frass (Coluccio et al., 2008), but to our knowledge, this is the first evidence of live vegetative cells in the larval gut. Thus, biological interactions between live gut yeasts and their host flies are possible.

**Fig. 2.**
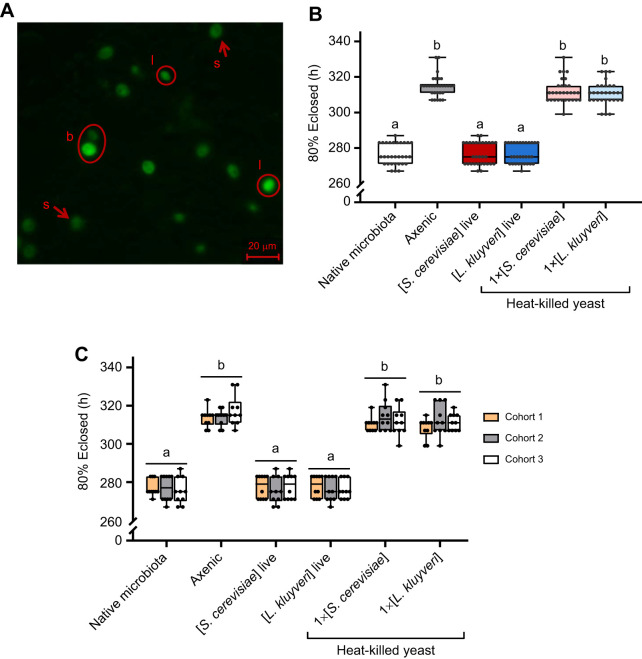
**Interaction with live yeasts reduces *Drosophila melanogaster* development time.** (A) Live yeast cells (GFP-*S. cerevisiae*) found in the gut content of *D. melanogaster* larva. Live cells are indicated by circles (l), one in the process of dividing (b, budding). Arrows point to digested yeast cells (s, spheroplasts). (B) Time at which 80% of the flies in each vial had eclosed (5 eggs per vial, 10 vials per treatment, 3 cohorts). Cohorts were pooled as there were no cohort effects in any of the treatments. (C) Time at which 80% of the flies in each vial had closed. Data are separated by cohorts (see Table S1 for statistics). Boxes represent the median and the 25th–75th percentiles; the whiskers represent the minimum and maximum values. Each dot represents a single vial. Significantly different groups by Tukey's HSD *post hoc* test are indicated by different letters. Data also presented as Mantel–Cox curves (Fig. S1, Table S2).

Presence or absence of gut microbes determined egg–adult development time. Flies reared with their native microbiota took an average of 276±6 h (ca. 11.5 days) for 80% of the flies in each vial to eclose; by contrast, axenic flies took more than a day longer (315±6 h) to develop (Fig. 2; Fig. S2). Either species of live yeast was sufficient to recover the native microbiota development time, and this accelerated development has also been reported by McMullen et al. (2020) and Mure et al. (2023) in *Drosophila* gnotobiotic for *H. uvarum*. There was no differential mortality among our treatment groups (cf. McMullen et al., 2020). Furthermore, providing dead yeast cells did not reduce development time in axenic flies (Fig. 2; Fig. S2), suggesting the yeasts must be alive to impact development time.

**Fig. 3.**
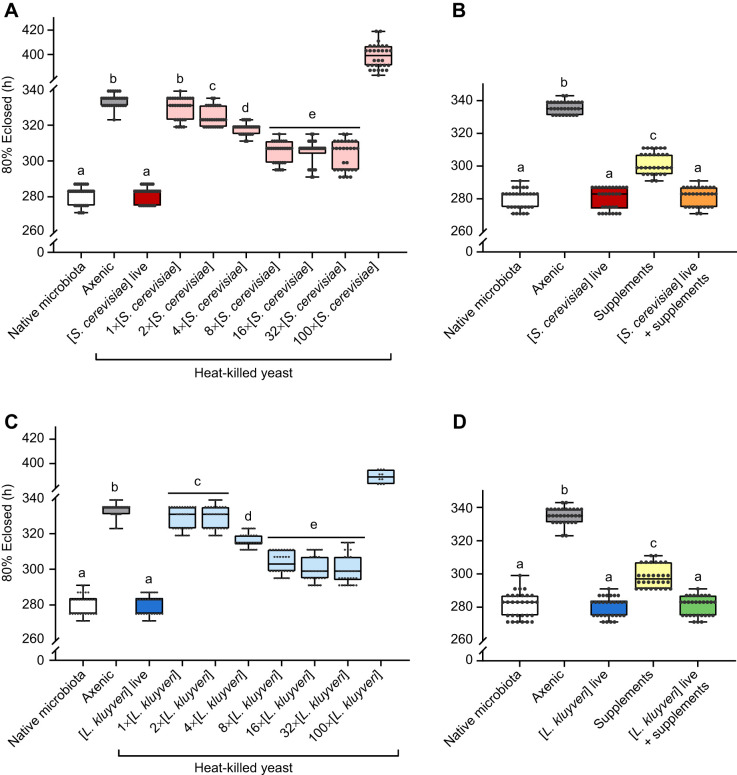
**The effect of yeasts on *D. melanogaster* development time is not purely nutritional.** Time at which 80% of the flies in each vial had eclosed (5 eggs per vial, 10 vials per treatment, 3 cohorts). Cohorts were pooled as there was no cohort effect in any of the treatments. (A) Effects of increasing amounts of heat-killed *S. cerevisiae* on *D. melanogaster* development time. (B) Effect of live *S. cerevisiae* and dietary supplements on *D. melanogaster* development time. The nutritional supplements are a mixture of 20 essential amino acids and six B-vitamins. (C) Effects of increasing amounts of heat-killed *L. kluyveri* on *D. melanogaster* development time. (D) Effect of live *L. kluyveri* and dietary supplements on *D. melanogaster* development time. Boxes represent the median and the 25th–75th percentiles; the whiskers extend to the minimum and maximum. Each point represents a single vial. Significantly different groups by Tukey's HSD *post hoc* test are indicated by different letters. Statistics are provided in Table S1.

**Fig. 4.**
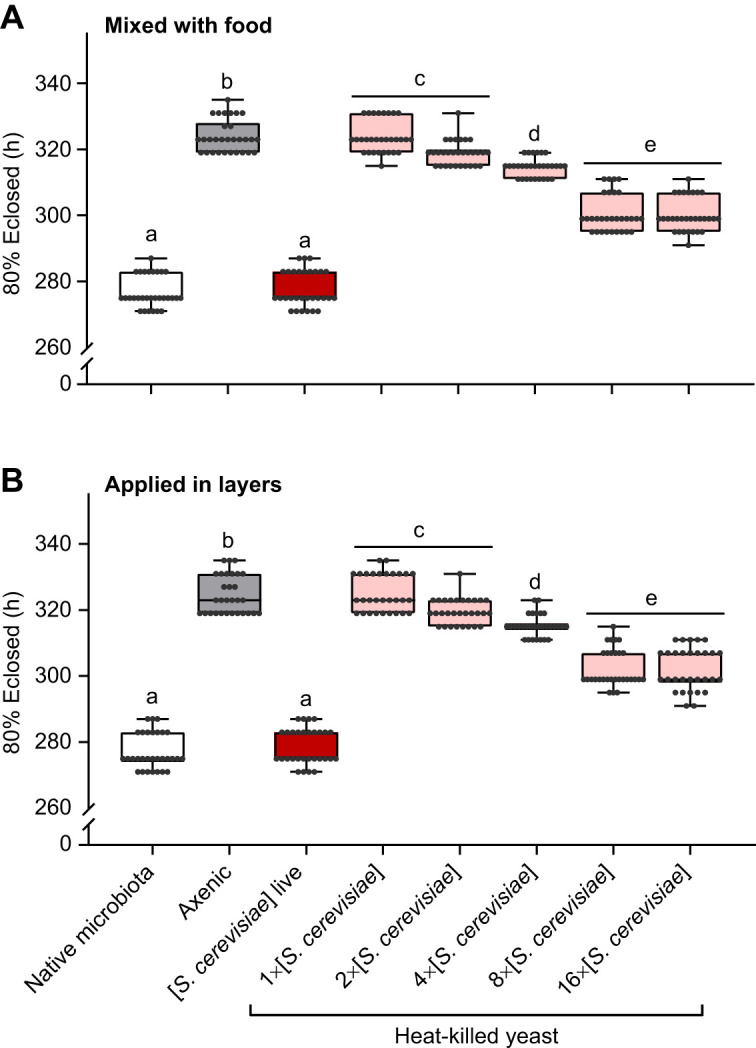
**Heat-killed yeast distributed throughout the food column affects *D.*** ***melanogaster*** **development time in the same manner as heat-killed yeast applied to the food surface.** (A) Live yeast suspension mixed with the food before dispensing and autoclaving. (B) heat-killed yeast suspension pipetted with each of seven 5 mm layers of sterile food. Boxes represent the median and the 25th–75th percentiles; the whiskers represent the minimum and maximum values. Each point represents a single vial (5 eggs per vial, 10 vials per treatment, 3 cohorts). Cohorts were pooled as there were no cohort effects in any of the treatments. Significantly different groups by Tukey's HSD *post hoc* test are indicated by different letters. Statistics are provided in Table S1.

Diet quality influences *Drosophila* development time (Wong et al., 2014; Krittika et al., 2019), and live yeasts grow (increasing the nutrient availability over time), whereas dead yeasts do not. Furthermore, larvae could deplete the nutrients available by consuming all the yeast from a single small addition (e.g. Keebaugh et al., 2019). We thus increased the quantity of dead yeast cells available to axenic flies, and large amounts of heat-killed yeast (8–32× the number of live yeast cells in our gnotobiotic treatments) reduced the development time difference between axenic and gnotobiotic flies by approximately 58% (Fig. 3), consistent with the accelerated development seen with high concentrations of heat-killed yeast by Mure et al. (2023). However, very large amounts of dead yeast (100× gnotobiotic) were detrimental: these flies took nearly 3 days longer to develop than their axenic counterparts (Fig. 3). Mixing dead cells into the food (cf. Mure et al., 2023) or layering the dead yeasts to make the nutrients available to larvae burrowed into the food did not further change development time (Fig. 4). Thus, although an effect of dead yeast suggests a macronutritional component to the influence of yeasts on development time, an interaction between live yeasts and the fly is necessary to completely explain the fast development of gnotobiotic flies. Previous work concluding that the influence of yeast is purely nutritional (e.g. Anagnostou et al., 2010; McMullen et al., 2020; Mure et al., 2023) may have missed the impact of live yeasts in the microbiota, especially if they did not compare live and heat-killed yeasts side-by-side in the same experiment.

Heat treatment disrupts the cell membranes, killing the yeast and making nutrients available outside the cell (Mogren et al., 1973; Mure et al., 2023). However, heat-killing may also inactivate some vitamins (Ottaway, 1993; Wang et al., 2004) or otherwise reduce the quality of the diet. Furthermore, higher concentrations of yeasts require longer heat exposure to kill all cells, increasing the risk of micronutrient degradation (note that Mure et al. 2023 used 80°C for 30 min, which may have yielded heat-killed yeast with different properties to ours). To account for any heat degradation of nutrients, we supplemented the diet of axenic flies with a sterile solution of 20 essential amino acids and six B-vitamins at concentrations previously used for supplementing *Drosophila* diets (Toshima and Tanimura, 2012; Wong et al., 2014). These nutritional supplements reduced development time, but only to the same extent as the heat-killed yeast treatments, and adding nutritional supplements to live yeast did not further reduce development time (Fig. 3). These effects of micronutritional supplements on *D. melanogaster* development time are broadly consistent with those reported elsewhere (Shin et al., 2011; Storelli et al., 2011; Wong et al., 2014; McMullen et al., 2020; Mure et al., 2023), and suggest that the impact of heat-killed yeast on development rate is nutritional. Indeed, Mure et al. (2023) found that heat-killed cells from yeasts that did not normally affect development could accelerate development, suggesting that access to cell contents is a primary difference among the species' effects. However, our nutritional treatments did not fully rescue the effects of the native microbiota on fly development.

We identify four important ways in which our methods differ from those of McMullen et al. (2020) and Mure et al. (2023), and that could explain why we differ from those studies by concluding that gut yeasts impact development time by both nutritional and non-nutritional mechanisms. First, we used a more recently collected (and outbred) population of *D. melanogaster*, compared with McMullen et al. (2020) and Mure et al. (2023), who used Canton-S flies, which are inbred (but show marked among-line variation; Colomb and Brembs, 2015) and have been in culture since before the 1940s (Stern, 1943), as well as different yeast species. Given the variation Mure et al. (2023) saw among yeast species, different fly lines and yeast species could yield markedly different results. Second, there may be significant differences in the production and culturing of axenic flies. Both McMullen et al. (2020) and Mure et al. (2023) used dechorionated embryos and Bloomington food, whereas we did not dechorionate our embryos and used the less-nutritious Tuscon stock center recipe (Markow and O'Grady, 2006), which we found performed better with autoclaving. However, although both McMullen et al. (2020) and Mure et al. (2023) reported high mortality (in fact, axenic flies did not grow at all in Mure et al.’s experiments), we had very little mortality, even in our axenic animals. Third, we included live yeast, axenic and native microbiota as controls in all of our experiments, which allowed us to directly compare the impact of a given treatment with the native microbiota; possibly this allowed us to recognize that heat-killed yeast did not fully rescue the native microbiota phenotype. Finally, we cultured samples from every one of our vials to identify any contamination, whereas Mure et al. (2023) discarded vials only when there was visible mold. It is possible that unseen contamination by bacteria or other yeast species could affect results, allowing the heat-killed yeast treatments to appear similar to controls. To further interrogate our conclusion that yeasts act as live symbionts, not just food, and perhaps to shed light on why our observations differ from other studies, we dissected the nature and timing of yeast effects on larval growth.

### Live yeasts accelerate larval development

**Fig. 5.**
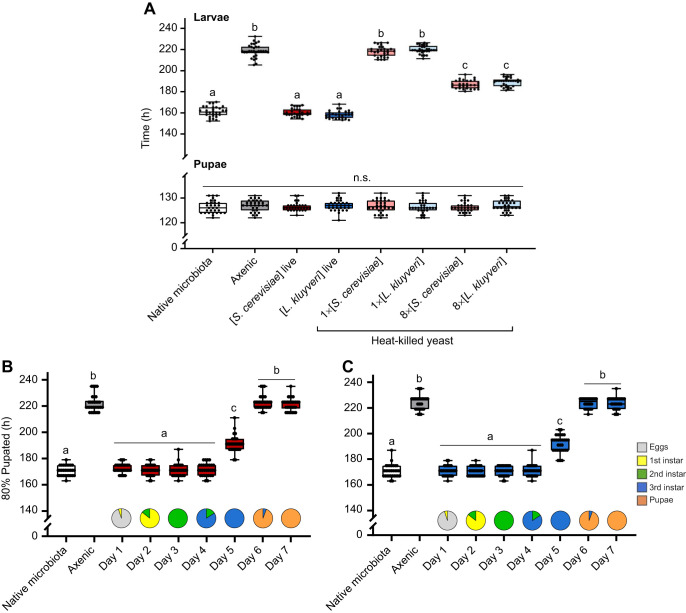
**Live yeasts decrease development time by accelerating larval development.** (A) Effect of live yeasts on time to pupariation (larvae) and duration of pupation (pupae, time elapsed from pupae formation to eclosion). Each point represents the mean time for each vial (10 vials per treatment, three cohorts). Although time to pupariation was significantly different (*P*<0.001), the duration of pupation was not different among treatments (*P*=0.867). (B) Effect of live *S. cerevisiae* on *D. melanogaster* pupariation time. (C) Effect of live *L. kluyveri* on *D. melanogaster* pupariation time. Cohorts were pooled as there was no cohort effect in any of the treatments. Day 1 is the day when the eggs were collected. A subset of the vials was then inoculated every 24 h, represented here by the consecutive numbered days. Boxes represent the median and the 25th–75th percentiles; the whiskers represent the minimum and maximum values. Each point represents a single vial (5 eggs per vial, 10 vials per treatment, 3 cohorts). Significantly different groups by Tukey's HSD *post hoc* test are indicated by different letters. Statistics are provided in Table S1.

**Fig. 6.**
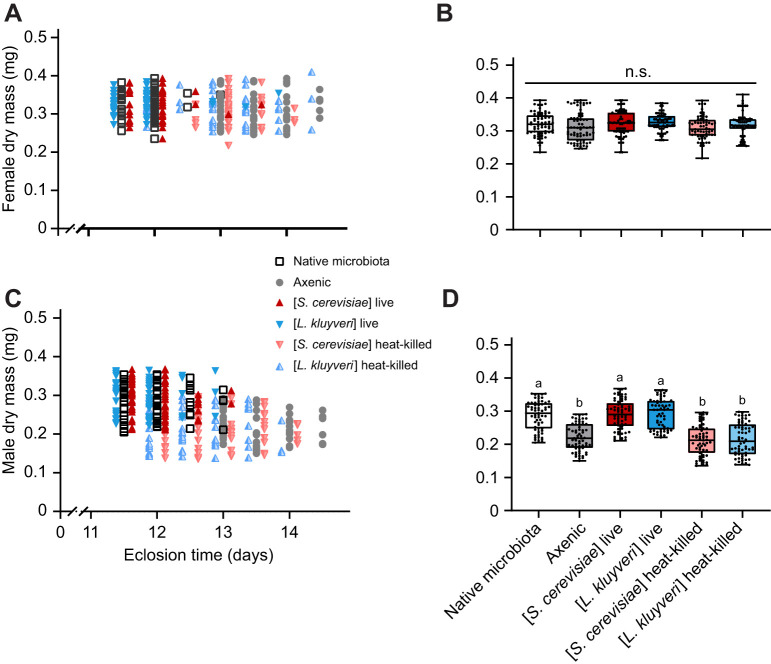
**Live yeasts accelerate larval development without body size trade-off.** Relationship between development time and dry mass in females (A) and males (C). Pearson correlation coefficients show no significant correlation between mass and eclosion time in any treatment (Table S3). Dry mass of adult females (B) and males (D). Boxes represent the median and the 25th–75th percentiles; the whiskers represent the minimum and maximum values. Each dot represents an individual fly (*n*=59–68 flies per treatment). One-way ANOVA (females *P*=0.069, males *P*<0.001); significantly different groups by Tukey's HSD *post hoc* test are indicated by different letters.

Holometabolous larvae accumulate nutrients to fuel metamorphosis and post-eclosion activities. We found that live yeasts reduced larval development time in gnotobiotic flies by 27% compared with their axenic counterparts, without changing pupation duration (Fig. 5A) or modifying adult (female) dry mass (Fig. 6B). Thus fast larval development in gnotobiotic flies was accompanied by fast growth. By contrast, axenic male adults (and those reared with heat-killed yeasts) were significantly smaller than their gnotobiotic counterparts (Fig. 6D), and consistent with McMullen et al. (2020), there was no relationship between development time and adult mass (Fig. 6). This contrasts with a general trade-off of fast larval development time for adult dry mass in *Drosophila* (Nunney, 1996; Chippindale et al., 1997). We conclude that live yeasts accelerate development by accelerating larval growth without reducing adult size, and speculate that the critical weight for pupariation likely remains unchanged. Strikingly, monocultures of either *S. cerevisiae* or *L. kluyveri* are sufficient to fully rescue the native microbiota development phenotype. This is consistent with other studies where the addition of yeasts rescued development phenotypes in the absence of other microbes (Anagnostou et al., 2010; Grangeteau et al., 2018; McMullen et al., 2020; Mure et al., 2023). However, whereas those authors interpreted this impact as macronutritional, we show that yeast macronutrients alone (heat-killed yeast cells) do not rescue the fast-developing phenotype.

**Fig. 7.**
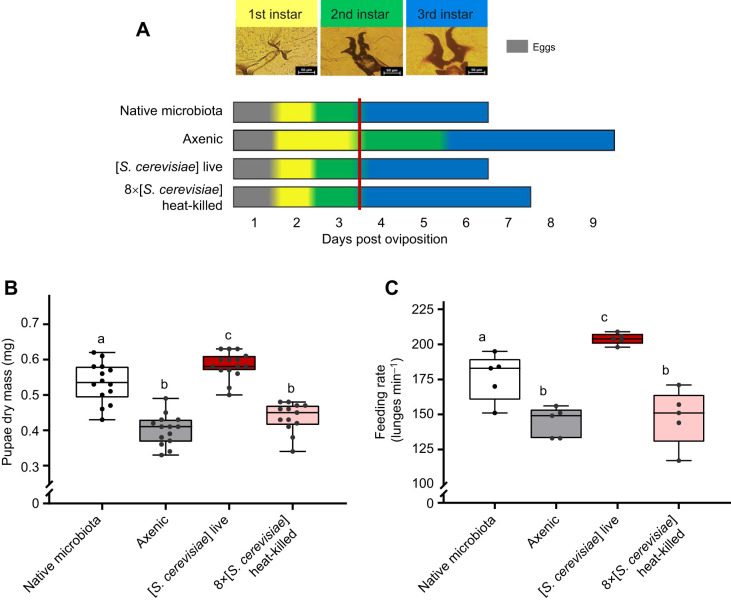
**Live yeast accelerates larval development, and increases third instar larval feeding rate and pupal mass.** (A) Duration of *D. melanogaster* larval stages in different treatments. Larval instars were determined by the morphology of mouth hooks and spiracles. The red vertical line indicates the time after which live yeast does not influence development time. Bars end at pupation. (B) Dry mass of pupae (*n*=13–15 pupae per treatment; *F*_3,52_=49.77, *P*<0.001). (C) Feeding rate of third instar larvae, determined by the number of mouth hook extensions (lunges) per minute. Each point represents a single vial (average feeding rate of three larvae per vial; five vials per treatment; *F*_3,16_=19.06, *P*<0.001). Boxes represent the median and the 25th–75th percentiles; the whiskers extend to the minimum and maximum. Significantly different groups by Tukey's HSD *post hoc* test are indicated by different letters.

When do yeasts accelerate larval development? We added live yeast to a subset of axenic larvae at 24 h intervals over 7 days. Larvae in vials inoculated with live yeasts during the first 4 days of larval development developed at the same rate as those with a native microbiota from the start (Fig. 5). However, providing yeast on day 5 post-oviposition added 20 h (12%) to development, and adding yeast on days 6 and 7 had no effect on development time (Fig. 5). The critical weight for metamorphosis in *Drosophila* is set late in the second instar (Bakker, 1959; Zhou et al., 2004). Axenic larvae develop more slowly than gnotobiotic larvae through the second and third instars: they molt from second to third instar 5 or 6 days post-oviposition, whereas all native microbiota and gnotobiotic larvae have entered the third instar by day 4 (Fig. 7A). However, providing axenic larvae with live yeasts before they molt to the third instar (i.e. before the critical weight is set), then their time to pupation is accelerated, rescuing the fast development time phenotype (Fig. 5).

We believe that the effect of yeasts on growth in the first two instars is primarily supported by the macro- and micro-nutrition gained from digesting the yeast cells [see McMullen et al. (2020) and Mure et al. (2023) for discussion of potential mechanisms underlying this effect]. Notably, development time of larvae provided with a high concentration of heat-killed yeast is midway between that of their axenic and gnotobiotic/native microbiota counterparts (Fig. 5). These larvae grow at a similar rate to their gnotobiotic and native microbiota counterparts during the first two instars, but growth slows in the third instar (Fig. 7A). Thus, live yeasts appear to specifically accelerate growth and development in the third larval instar, when holometabolous insect larvae gain most of their mass (De Moed et al., 1999; Nijhout et al., 2014). Furthermore, because mass gain accelerates during the final instar (Mirth et al., 2005), the requirement of live yeasts when molting into the third instar does not coincide with the period of greatest nutrient demand (towards the end of the third instar). Finally, when development is accelerated by live yeasts, there is no impact on pupal size (Fig. 7B). Therefore, the influence of yeasts is not a result of adding yeast nutrients per se, but rather appears to result from a change in the biology of the larva.

We identify four hypotheses that could explain the impact of yeasts on larval growth. First, metabolic products of yeasts might accelerate growth. Although our nutrient supplement treatments did partially speed development, this acceleration was confined to the first two instars (Fig. 7A). Thus, although the macro- or micro-nutritional contribution of yeasts early in development is consistent with nutritional contributions of other gut microbes (Shin et al., 2011; Qiao et al., 2019; Schmidt and Engel, 2021), it does not persist in the third instar, when most growth occurs. Mure et al. (2023) specifically identify the branched-chain amino acids leucine and isoleucine as yeast products that might mediate developmental acceleration; however, adding nutrient supplements that include these molecules did not accelerate larval development (Fig. 3).

Second, yeasts could directly interact with host pathways associated with regulating growth. Insulin signaling pathways have an overarching influence on growth and development in insects (Biglou et al., 2021), so we tested whether mutants associated with insulin receptor binding stopped growing faster when provided with live yeasts. Deleting the primary insulin-like peptide *Ilp-2* did not prevent accelerated development in response to native microbiota or live yeasts, but these mutants did not accelerate growth in response to 8× heat-killed yeasts as we observed in wild-type flies (Fig. S2). By contrast, flies underexpressing the *IRS-*ortholog *chico* accelerated development in response to native microbiota, live yeasts and 8× heat-killed yeast, which is consistent with the responses of wild-type flies to these treatments (Fig. S2). This suggests that live yeasts do not directly influence growth via insulin signaling, but that insulin signaling is required for macronutrient-related growth early in development. Not all pathways downstream of the insulin receptor Inr are mediated by CHICO (Werz et al., 2009), so we cannot rule out yeast interactions with other components of insulin signaling (Biglou et al., 2021), nutrient sensing (such as TORC1; see Mure et al., 2023) or other development-regulating pathways (Layalle et al., 2008; Cao et al., 2022). Further investigation of the direct roles of yeasts will benefit from extensive screening of *Drosophila* mutants, beyond the scope of this study.

Third, the microbiome–gut–brain axis could change the behavior of gnotobiotic larvae, such that they consume more food. Third instar larvae reared with live *S. cerevisiae* had a higher feeding rate with lower variance than all other groups, including those with the native microbiota (Fig. 7C). Although larvae reared with their native microbiota developed at the same time as larvae with live yeast, they fed more slowly. Thus, increased feeding rate (assuming digestion can keep up) could partially account for the effect of live yeasts on development (also discussed by McMullen et al., 2020).

And fourth, live yeasts may initiate gut remodeling that improves nutrient absorption and/or digestive efficiency. Such remodeling can accelerate growth and development in response to gut bacteria (Shin et al., 2011; Broderick et al., 2014). Although beyond the scope of the present study, this hypothesis could be tested by examining gut ultrastructure (Broderick et al., 2014), measuring food conversion efficiency (Slansky and Wheeler, 1989), and identifying the receptors associated with yeast–gut interaction – perhaps beginning with receptors that detect yeasts and other fungi as part of the immune response (e.g. Matskevich et al., 2010).

### Concluding remarks

Yeasts are integral to the *D. melanogaster* gut microbiome. We show that yeasts not only provide nutrition as part of a meal, but interact beneficially with the fly as gut symbionts. Although yeasts contribute nutrition during early growth, only living yeasts trigger faster feeding and development in the third larval instar. Thus, the macro- and micro-nutritional contribution of yeast can be conflated with its biotic interactions, implying that gut microbiome studies that do not account for yeasts could miss symbiotic yeast–host relationships. This reduced emphasis on yeasts almost certainly extends beyond *Drosophila*. One reason for this is that (prevalent) 16S metagenomic studies do not detect yeasts (Forbes et al., 2019), but we also show here that the macronutritional effects of yeast must be clearly teased apart from their potential biological interactions. Yeasts clearly contribute to the microbiota of almost all taxa, including vertebrates (Sokół et al., 2018; Lai et al., 2019), and we speculate that yeasts could interact with the microbiota–gut–brain axis in many animals.

We show here that yeasts speed development time in a manner that likely increases fitness in *Drosophila.* Because flies acquire their gut yeasts from the environment, there is geographic and seasonal variation in the species and strain found in insect guts in nature (Chen et al., 2016; Chakraborty et al., 2020). Given the size of the effect on development time, it is plausible that plasticity of some traits in nature could derive from yeast–host insect interactions such as the one we describe. But what is the benefit for the yeast? Insects and yeasts have co-evolved (Starmer et al., 1991; Becher et al., 2018), and although yeasts might benefit simply from dispersal (Starmer and Fogleman, 1986), recent work suggests that the insect host's gut may be the location of yeast sexual reproduction and sporulation (Stefanini et al., 2016). Thus, the gut yeast–host insect interaction has the potential to be a complex and rich mutualistic relationship, not simply a source of food to the insect and dispersal for the yeast.

## Supplementary Material

10.1242/jexbio.247932_sup1 Supplementary information

Dataset 1. Excel workbook containing all data presented in the paper.
