# Supplementary material for: Live yeasts accelerate Drosophila melanogaster larval development
Source: J Exp Biol. 2024 Oct 4;227(19):jeb247932. doi: 10.1242/jeb.247932 (PMC11463955; doi:10.1242/jeb.247932)
Supplement: Supplementary information [file jexbio-227-247932-s1.pdf]

Journal of Experimental Biology • Supplementary information

Supplementary Materials and Methods

Assessing axenic state

We also used PCR to detect any bacterial or archaeal DNA in putatively axenic flies. Briefly, we homogenized individual flies from two new axenic cohorts prepared on consecutive days (n= one fly per vial, five vials for cohort 1 and six for cohort 2) in 220 µL of sterile water. We plated 10 µl of each homogenate and 10 µl of homogenized food (c. 3 µl of food scraped from the surface of each vial homogenized in 100 µl of sterile water) on both YM agar and LB agar (1 % Tryptone, 0.5 % yeast extract, 1.5 % agar) to identify any visible microbial growth. We extracted DNA from the remaining homogenate using a Phenol:Chloroform protocol described previously (Lachance *et al.* 2016). Each PCR reaction contained 5.0 µL Template and a final concentration of 1X PCR Buffer, 0.2 mM (each) dNPT mixture, 1.5 mM $MgCl_2$, 0.2 µM (each) primer mix, 1.0 unit Taq DNA Polymerase (Taq Platinum, Invitrogen, ThermoFisher, Burlington, ON, Canada). We used 35 cycles with denaturation at 92 °C for 10 s, annealing at 53 °C for 15 s (decreasing by 0.1°C/cycle), and extension at 72 °C for 20 s (increasing by 1 s/ cycle). We used the 16S rRNA primers Pro341F: 5'-CCTACGGGNBGCASCAG-3' and Pro805R: 5'-GACTACNVGGGTATCTAATCC-3' (Takahashi *et al.* 2014). We ran the PCR reaction product on an agarose gel and identified a single band in two samples from different vials in cohort 1 (from a total of 12 vials across two cohorts (Jimenez-Padilla 2016)). We excised this band and sequenced it (Sanger Sequencing, London Regional Genomics Centre, London, ON, Canada). The resulting sequence aligned to *Staphylococcus epidermidis*. *Staphylococcus epidermidis* is associated with human skin (Otto 2009), is not normally associated with *Drosophila* (Chandler *et al.* 2011), and the bands were present in only one cohort. The YM agar plates from these samples each had one colony forming unit (CFU), but the food samples from these vials yielded no colonies when cultured at 25 °C for ten days. We believe that these two instances likely arose from human skin contamination of the samples during the processing of axenic flies after rearing rather than bacterial growth in the vials or the flies. Thus, we are confident that our method generates axenic flies.

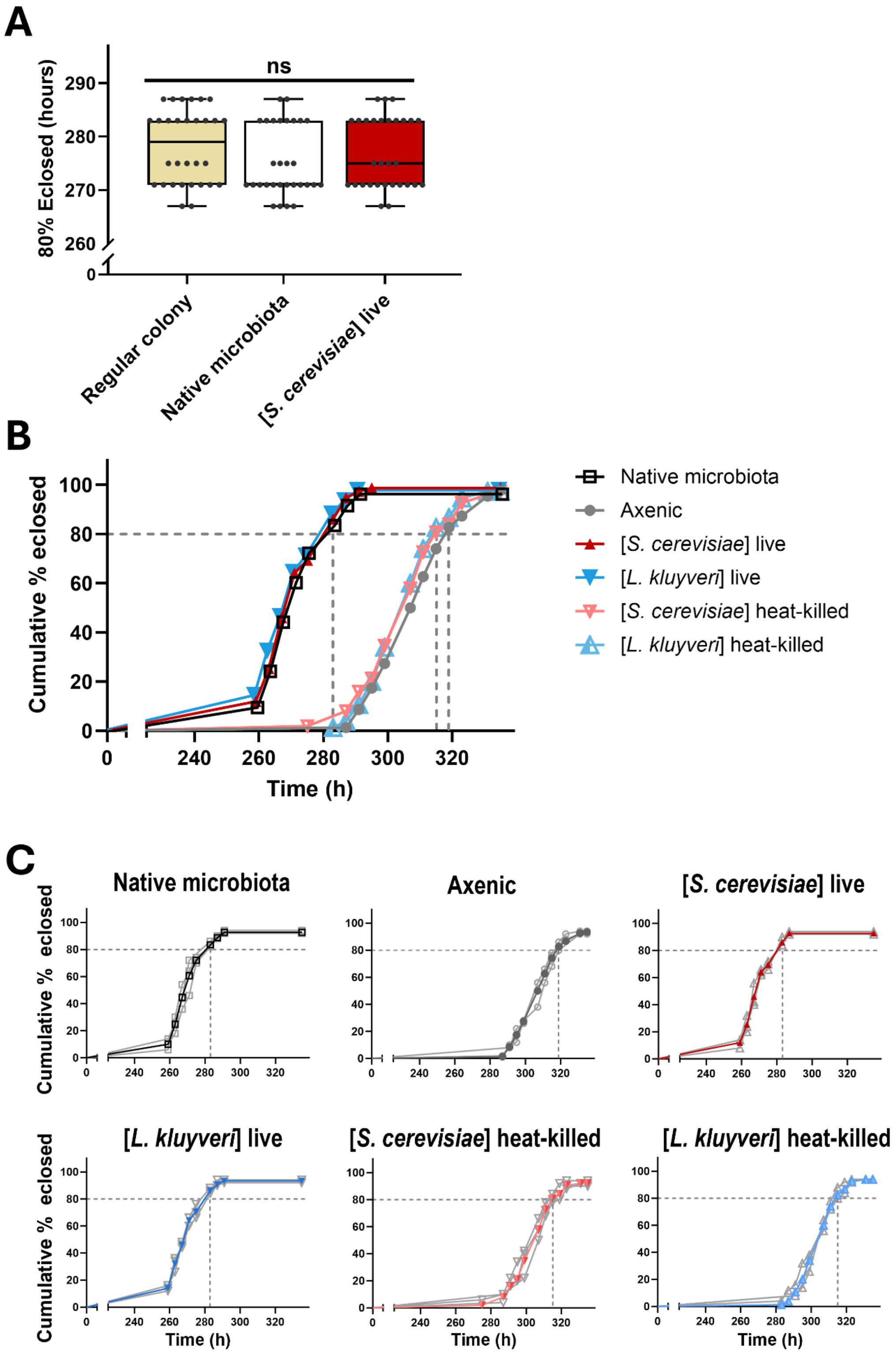

Journal of Experimental Biology • Supplementary information

Fig. S1. Effects of microbes on *Drosophila melanogaster* development.
A. Flies reared in the native microbiota treatment developed as fast as the flies from our conventional rearing. For the conventional colony flies, we placed the eggs directly on sterile yeast-sugar agar and transferred them to standard Tucson food. The parents are the same for all treatments in each cohort (five eggs/vial, ten vials/treatment, three cohorts, sexes not separated). Cohorts were pooled as there were no cohort effects in any of the treatments (see Table S1). Boxes represent the median and the 25th – 75th percentile; the whiskers extend to the minimum and maximum. Each point represents a single vial. Eclosion time did not differ significantly among treatments (P = 0.242; statistics in Table S1).

B. Live yeasts consistently change the shape of *D. melanogaster* cumulative development in each cohort. Mantel-Cox curves including all data points presented in Figs. 2 (five eggs/vial, ten vials/treatment, three cohorts pooled). Horizontal dashed line is at 80%; vertical dashed lines indicate time to 80% eclosion calculated from the pooled data and presented in Fig. 2. Data were pooled for presentation and analysis in the main manuscript. Statistics are provided in Table S2.

C: Data in B separated by cohort. Three cohorts for each treatment (grey lines) colored lines are the pooled data presented in B. Cohorts did not differ within any treatment [Mantel-Cox Log-Rank test followed by a Benjamini-Hochberg False Discovery Rate (FDR) correction for multiple comparisons]. Statistics are provided in Table S2.

Journal of Experimental Biology • Supplementary information

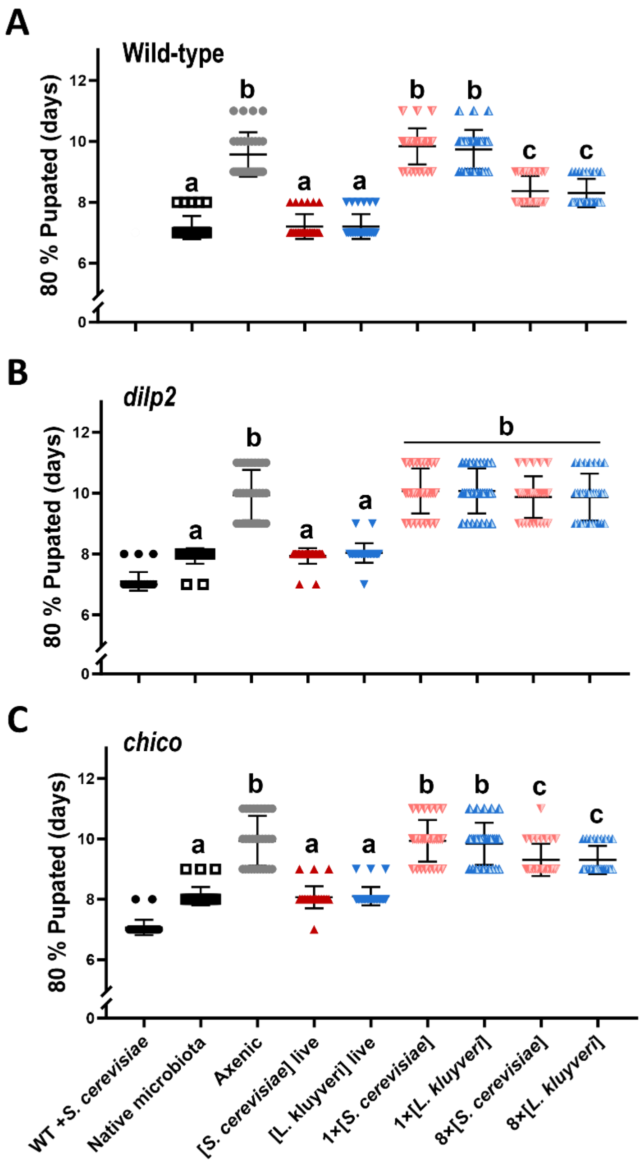

Fig. S2. Two genes in the insulin-like signaling pathway do not mediate the effect of live yeasts on *D. melanogaster* larval development. Time at which 80 % of the flies in each vial had pupated. (**A**) Pupation time in days of wild-type (WT) *D. melanogaster*. Data also presented in hours in Fig. 5A. (**B**) Pupation time of a *dilp2* knockout mutant. (**C**) Pupation time of a *chico* hypomorphic mutant. The WT+*S. cerevisiae* group was included during experiments to ensure mutant *Drosophila* lines were tested under the same conditions as throughout the study but were not included in the statistical analysis (wild-type: $W_{7,98}$=121.5, P<0.001; *dilp2*: $W_{7,99}$=85.75, P<0.001; *chico*: $W_{7,99}$=144.6, P<0.001). The mean and standard deviation are shown; symbols represent single vials (five eggs/vial, n=ten vials/treatment, three cohorts). Significantly different groups are indicated by different letters (Dunnett's T3 multiple comparisons test for unequal variance).

Journal of Experimental Biology • Supplementary information

Table S1. Summary statistics for data presented in the main text, both with all three cohorts pooled (one-way ANOVA) and also with the data separated by cohort (two-way ANOVA). Terms in bold typeface indicate statistical significance.

Figure	Pooled Data	Separated by Cohort	
**2B & 2C**	**$F_{5,174}$=309.6; P<0.001**	***Treatment***	**$F_{5,162}$=305.1; P<0.001**
		Cohort	$F_{2,162}$=0.462; P=0.631
		Interaction	$F_{10,162}$=0.857; P=0.575
**3A**	**$F_{8,261}$=350.3; P<0.001**	***Treatment***	**$F_{8,243}$=350.6; P<0.001**
		Cohort	$F_{2,243}$=0.072; P=0.930
		Interaction	$F_{16,243}$=1.130; P=0.328
**3B**	**$F_{4,145}$=511.8; P<0.001**	***Treatment***	**$F_{4,135}$=496.7; P<0.001**
		Cohort	$F_{2,135}$=0.626; P=0.536
		Interaction	$F_{8,135}$=0.558; P=0.810
**3C**	**$F_{8,261}$=316.8; P<0.001**	***Treatment***	**$F_{8,243}$=306.9; P<0.001**
		Cohort	$F_{2,243}$=0.095; P=0.909
		Interaction	$F_{16,243}$=0.601; P=0.882
**3D**	**$F_{4,145}$=426.5; P<0.001**	***Treatment***	**$F_{4,135}$=412.9; P<0.001**
		Cohort	$F_{2,135}$=0.945; P=0.391
		Interaction	$F_{8,135}$=0.434; P=0.899
**4A**	**$F_{7,232}$=494.0; P<0.001**	***Treatment***	**$F_{7,216}$=468.3; P<0.001**
		Cohort	$F_{2,216}$=0.646; P=0.525
		Interaction	$F_{14,216}$=0.500; P=0.932
**4B**	**$F_{7,232}$=398; P<0.001**	***Treatment***	**$F_{7,216}$=394.8; P<0.001**
		Cohort	$F_{2,216}$=1.840; P=0.163
		Interaction	$F_{14,216}$=0.744; P=0.729
**5A (Larvae)**	**$F_{7,232}$=1120; P<0.001**	***Treatment***	**$F_{7,216}$=1090; P<0.001**
		Cohort	$F_{2,216}$=0.702; P=0.497
		Interaction	$F_{14,216}$=0.591; P=0.871
**5A (Pupae)**	$F_{7,232}$=1.467; P=0.867	*Treatment*	$F_{7,216}$=0.633; P=0.728
		Cohort	$F_{2,216}$=3.017; P=0.051
		Interaction	$F_{14,216}$=0.514; P=0.924
**5B**	**$F_{8,261}$=756.2; P<0.001**	***Treatment***	**$F_{8,243}$=727.2; P<0.001**
		Cohort	$F_{2,243}$=0.117; P=0.889
		Interaction	$F_{16,243}$=0.486; P=0.953
**5C**	**$F_{8,261}$=913.4; P<0.001**	***Treatment***	**$F_{8,243}$=903.7; P<0.001**
		Cohort	$F_{2,243}$=0.228; P=0.797
		Interaction	$F_{16,243}$=0.923; P=0.543
**S1A**	$F_{2,87}$=1.44; P=0.242	*Treatment*	$F_{2,81}$=1.387; P=0.256
		Cohort	$F_{2,81}$=0.072; P=0.930
		Interaction	$F_{4,81}$=0.644; P=0.633

Journal of Experimental Biology • Supplementary information

Table S2. Live yeasts consistently change the shape of *D. melanogaster* cumulative development in each cohort. Summary statistics for Fig. S1B and S1C. The Mantel-Cox curves include all eclosion times per vial (n=47-50 flies/treatment/cohort) complementary to the 80% eclosion times presented in Fig. 2. Table-wide FDR correction did not alter significance for cohort comparisons. Unadjusted values shown. Terms in bold typeface indicate statistical significance.

Figure	Statistic	*P*
**Pooled data**	***df*=5, χ^2=631.7**	**<0.001**
Cohorts		
Native microbiota	*df*=2, χ^2=0.78	0.677
Axenic	*df*=2, χ^2=1.14	0.566
[S. cerevisiae] live	*df*=2, χ^2=0.12	0.943
[L. kluyveri] live	*df*=2, χ^2=0.11	0.945
[S. cerevisiae] heat-killed	*df*=2, χ^2=3.27	0.195
[L. kluyveri] heat-killed	*df*=2, χ^2=0.82	0.664

Table S3. Correlation between development time and adult dry mass. Pearson correlation coefficients for Fig. 6. *P* (one-tailed), α = 0.05; n=61-68 flies/treatment. Table-wide FDR correction did not alter significance for any treatment or sex; Unadjusted values shown. Terms in bold typeface indicate statistical significance.

Treatment	Females		Males	
	r	***P***	***r***	***P***
Native microbiota	0.06	0.317	-0.09	0.248
Axenic	-0.04	0.375	-0.18	0.084
[*S. cerevisiae*] live	0.02	0.432	-0.19	0.069
[*L. kluyveri*] live	0.13	0.161	0.05	0.361
[*S. cerevisiae*] heat-killed	-0.04	0.394	0.02	0.439
[*L. kluyveri*] heat-killed	0.01	0.484	-0.004	0.488

Dataset 1. Excel workbook containing all data presented in the paper.

Available for download at
https://journals.biologists.com/jeb/article-lookup/doi/10.1242/jeb.247932#supplementary-data

Journal of Experimental Biology • Supplementary information

SI References

Chandler, J.A., Lang, J.M., Bhatnagar, S., Eisen, J.A. and Kopp, A. (2011). Bacterial communities of diverse *Drosophila* species: ecological context of a host-microbe model system. *PLoS Genetics* **7**, e1002272.

Jiménez-Padilla, Y. (2016). Effect of gut-associated yeasts on *Drosophila melanogaster* performance. *MSc Thesis* University of Western Ontario, Londopn, ON, Canada.

Lachance, M.-A., Hurtado, E. and Hsiang, T. (2016). A stable phylogeny of the large-spored *Metschnikowia* clade. *Yeast* **33,** 261-275.

Otto, M. (2009). *Staphylococcus epidermidis* — the 'accidental' pathogen. *Nature Reviews Microbiology* **7**, 555-567.

Takahashi, S., Tomita, J., Nishioka, K., Hisada, T. and Nishijima, M. (2014). Development of a Prokaryotic Universal Primer for Simultaneous Analysis of Bacteria and Archaea Using Next-Generation Sequencing. *PLoS ONE* **9**, e105592.

Journal of Experimental Biology • Supplementary information
